# 3-(Adamantan-1-yl)-4-ethyl-1-{[4-(2-meth­oxy­phen­yl)piperazin-1-yl]meth­yl}-1*H*-1,2,4-triazole-5(4*H*)-thione

**DOI:** 10.1107/S1600536813032789

**Published:** 2013-12-07

**Authors:** Ali A. El-Emam, Hanaa M. Al-Tuwaijri, Ebtehal S. Al-Abdullah, C. S. Chidan Kumar, Hoong-Kun Fun

**Affiliations:** a Department of Pharmaceutical Chemistry, College of Pharmacy, King Saud University, PO Box 2457, Riaydh 11451, Saudi Arabia; b X-ray Crystallography Unit, School of Physics, Universiti Sains Malaysia, 11800 USM, Penang, Malaysia

## Abstract

In the title compound, C_26_H_37_N_5_OS, the piperazine ring adopts a chair conformation. The triazole ring forms dihedral angles of 67.85 (9) and 59.41 (9)° with the piperazine and benzene rings, respectively, resulting in an approximate V-shaped conformation for the mol­ecule. An intra­molecular C—H⋯O hydrogen bond generates an *S*(6) ring motif. The crystal structure features C—H⋯π inter­actions, producing a two-dimensional supramolecular architecture.

## Related literature   

For the pharmacological activity of adamantane derivatives and adamantyl-1,2,4-triazoles, see: Togo *et al.* (1968 ▶); El-Emam *et al.* (2004 ▶, 2013 ▶); Al-Deeb *et al.* (2006 ▶); Kadi *et al.* (2007 ▶, 2010 ▶). For related adamantyl-1,2,4-triazole structures, see: Al-Abdullah *et al.* (2013 ▶); Al-Tamimi, Alafeefy *et al.* (2013 ▶); Al-Tamimi, Al-Abdullah *et al.* (2013 ▶); El-Emam *et al.* (2012 ▶). For the synthesis of the starting material, see: El-Emam & Ibrahim (1991 ▶). For ring conformations and ring puckering analysis, see: Cremer & Pople (1975 ▶). For hydrogen-bond motifs, see: Bernstein *et al.* (1995 ▶).
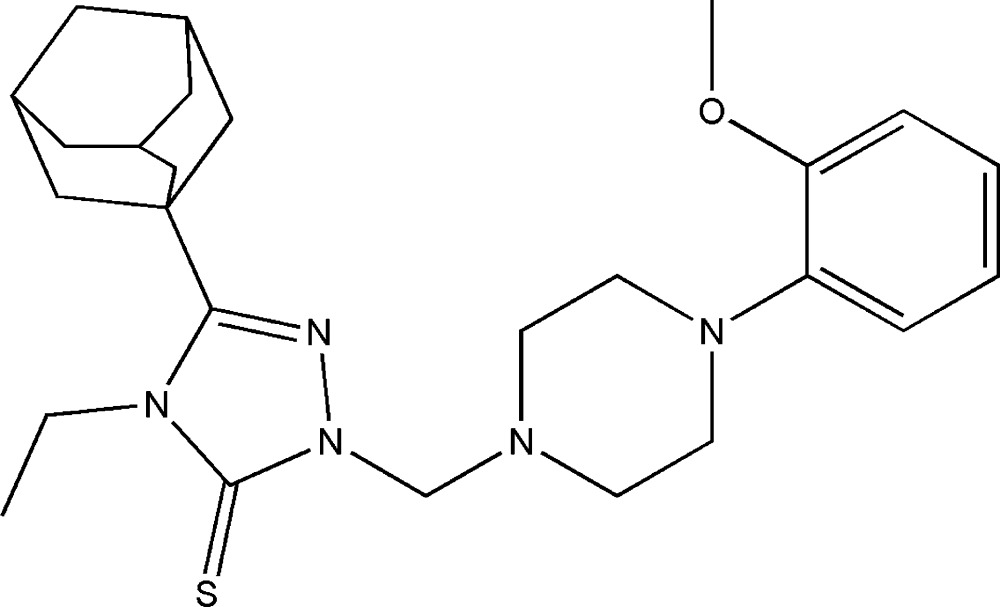


## Experimental   

### 

#### Crystal data  

C_26_H_37_N_5_OS
*M* *_r_* = 467.67
Monoclinic, 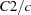

*a* = 19.8170 (3) Å
*b* = 11.9384 (3) Å
*c* = 21.7807 (4) Å
β = 107.886 (2)°
*V* = 4903.90 (17) Å^3^
*Z* = 8
Cu *K*α radiation
μ = 1.39 mm^−1^
*T* = 296 K
0.98 × 0.62 × 0.41 mm

#### Data collection  

Bruker APEXII CCD diffractometer
Absorption correction: multi-scan (*SADABS*; Bruker, 2009 ▶) *T*_min_ = 0.344, *T*_max_ = 0.599
15455 measured reflections
4029 independent reflections
3606 reflections with *I* > 2σ(*I*)
*R*_int_ = 0.033

#### Refinement  

*R*[*F* ^2^ > 2σ(*F* ^2^)] = 0.043
*wR*(*F* ^2^) = 0.115
*S* = 1.05
4029 reflections
308 parameters
H atoms treated by a mixture of independent and constrained refinement
Δρ_max_ = 0.19 e Å^−3^
Δρ_min_ = −0.27 e Å^−3^

### 

Data collection: *APEX2* (Bruker, 2009 ▶); cell refinement: *SAINT* (Bruker, 2009 ▶); data reduction: *SAINT*; program(s) used to solve structure: *SHELXTL* (Sheldrick, 2008 ▶); program(s) used to refine structure: *SHELXTL*; molecular graphics: *SHELXTL*; software used to prepare material for publication: *SHELXTL* and *PLATON* (Spek, 2009 ▶).

## Supplementary Material

Crystal structure: contains datablock(s) global, I. DOI: 10.1107/S1600536813032789/rz5099sup1.cif

Structure factors: contains datablock(s) I. DOI: 10.1107/S1600536813032789/rz5099Isup2.hkl

Supporting information file. DOI: 10.1107/S1600536813032789/rz5099Isup3.cml

Additional supporting information:  crystallographic information; 3D view; checkCIF report

## Figures and Tables

**Table 1:** Hydrogen-bond geometry (Å, °) *Cg* is the centroid of the C1–C6 benzene ring.

*D*—H⋯*A*	*D*—H	H⋯*A*	*D*⋯*A*	*D*—H⋯*A*
C11—H11*A*⋯O1	0.97	2.26	2.903 (2)	123
C18—H18*A*⋯*Cg* ^i^	0.97	2.81	3.748 (2)	162

Symmetry code: (i) 
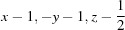
.

**Fig. 1.**
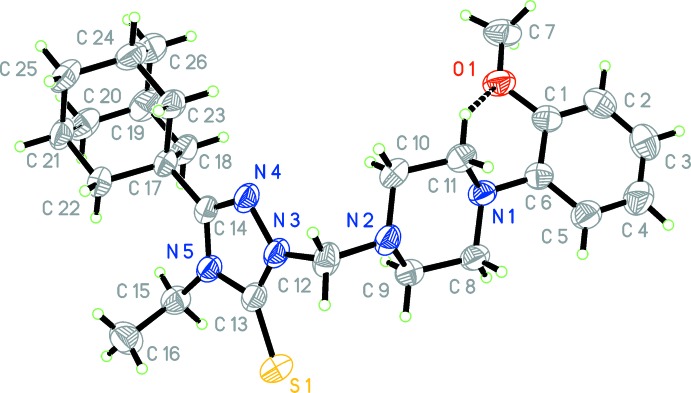
The molecular structure of the title compound with 50% probability displacement ellipsoids. The intramolecular hydrogen bond is shown as a dashed line.

## supplementary crystallographic information

### 1. Comment

Derivatives of adamantane have long been known for their diverse biological activities including antiviral activity against influenza (Togo *et al.*, 1968) and HIV viruses (El-Emam *et al.*, 2004). Moreover, adamantane derivative were reported to exhibit marked antibacterial and anti-inflammatory activities (Kadi *et al.*, 2007, 2010; El-Emam *et al.*, 2013). In continuation of our interest in the chemical and pharmacological properties of adamantane derivatives, and as part of our on-going structural studies of adamantane derivatives (Al-Abdullah *et al.*, 2013); Al-Tamimi, Alafeefy *et al.*, 2013; Al-Tamimi, Al-Abdullah *et al.*, 2013; El-Emam *et al.*, 2012), we have synthesized the title compound (I) as a potential chemotherapeutic agent.

In the crystal structure of the title compound (Fig. 1), the piperazine (N1–N2/C8–C11) ring adopts a chair conformation with puckering parameters: Q = 0.5783 (18) Å, θ = 178.03 (17)°, and φ = 25 (5)° (Cremer & Pople, 1975). The dihedral angle between the piperazine ring and the triazole ring (N3–N5/C13/C14) is 67.85 (9)°. The triazole ring forms a dihedral angle of 59.41 (9)° with the benzene ring (C1—C6), resulting in an approximate V-shape conformation of the molecule. An intramolecular C–H···O hydrogen bond generates an *S*(6) ring motif (Bernstein *et al.*, 1995). The crystal structure features an intermolecular C–H···π interaction with a H18A···*Cg* distance of 2.81 Å, where *Cg* is the centroid of the benzene ring (C1—C6).

### 2. Experimental

A mixture of 527 mg (2 mmol) of 3-(1-adamantyl)-4-ethyl-4*H*-1,2,4- triazole-5-thiol (El-Emam & Ibrahim, 1991), 1-(2-methoxyphenyl)piperazine (383 mg, 2 mmol) and 37% formaldehyde solution (1 ml) in ethanol (8 ml) was heated under reflux for 15 min until a clear solution was obtained. Stirring was continued for 12 h at room temperature and the mixture was allowed to stand overnight. Cold water (5 ml) was added slowly and the mixture was stirred for 20 min. The precipitated crude product were filtered, washed with water, dried, and crystallized from ethanol to yield 860 mg (92%) of the title compound (C_26_H_37_N_5_OS) as colourless needle crystals. M.p.: 477–479 K. Single plate-shaped crystals suitable for X-ray analysis were obtained by slow evaporation of a CHCl_3_:EtOH solution (1:1 *v*/*v*; 5 ml) at room temperature.

^1^H NMR (CDCl_3_, 500.13 MHz): δ 1.32 (t, 3H, CH_2_CH_3_, J = 7.0 Hz), 1.71–1.76 (m, 6H, Adamantane-H), 1.98–2.12 (m, 9H, Adamantane-H), 3.08 (s, 8H, Piperazine-H), 3.81 (s, 3H, OCH_3_), 4.15 (q, 2H, CH_2_CH_3_, J = 7.0 Hz), 5.15 (s, 2H, CH_2_), 6.79–7.01 (m, 4H, Ar—H). ^13^C NMR (CDCl_3_, 125.76 MHz): δ 13.76 (CH_2_CH_3_), 27.92, 35.32, 36.48, 39.83 (Adamantane-C), 43.83 (CH_2_CH_3_), 47.40, 50.18 (Piperazine-C), 55.48 (OCH_3_), 72.58 (CH_2_), 111.43, 118.38, 121.12, 123.55, 152.13, 152.26 (Ar—C), 156.57 (Triazole C-5), 167.34 (C=S).

### 3. Refinement

The H atoms bound to atom C12 were located in a difference Fourier map and refined freely. All other H atoms were positioned geometrically [C—H = 0.93–1.01 Å] and refined using a riding model with *U*_iso_(H) = 1.2 *U*_eq_(C) or 1.5 *U*_eq_(C) for methyl H atoms. A rotating group model was used for the methyl groups.

### Crystal data

**Table:**

C_26_H_37_N_5_OS	*F*(000) = 2016
*M**_r_* = 467.67	*D*_x_ = 1.267 Mg m^−^^3^
Monoclinic, *C*2/*c*	Cu *K*α radiation, λ = 1.54178 Å
Hall symbol: -C 2yc	Cell parameters from 4154 reflections
*a* = 19.8170 (3) Å	θ = 4.3–69.2°
*b* = 11.9384 (3) Å	µ = 1.39 mm^−^^1^
*c* = 21.7807 (4) Å	*T* = 296 K
β = 107.886 (2)°	Plate, colourless
*V* = 4903.90 (17) Å^3^	0.98 × 0.62 × 0.41 mm
*Z* = 8	

### Data collection

**Table:**

Bruker APEXII CCD diffractometer	4029 independent reflections
Radiation source: fine-focus sealed tube	3606 reflections with *I* > 2σ(*I*)
Graphite monochromator	*R*_int_ = 0.033
φ and ω scans	θ_max_ = 65.0°, θ_min_ = 4.3°
Absorption correction: multi-scan (*SADABS*; Bruker, 2009)	*h* = −22→23
*T*_min_ = 0.344, *T*_max_ = 0.599	*k* = −9→14
15455 measured reflections	*l* = −25→21

### Refinement

**Table:**

Refinement on *F*^2^	Primary atom site location: structure-invariant direct methods
Least-squares matrix: full	Secondary atom site location: difference Fourier map
*R*[*F*^2^ > 2σ(*F*^2^)] = 0.043	Hydrogen site location: inferred from neighbouring sites
*wR*(*F*^2^) = 0.115	H atoms treated by a mixture of independent and constrained refinement
*S* = 1.05	*w* = 1/[σ^2^(*F*_o_^2^) + (0.0627*P*)^2^ + 2.6908*P*] where *P* = (*F*_o_^2^ + 2*F*_c_^2^)/3
4029 reflections	(Δ/σ)_max_ < 0.001
308 parameters	Δρ_max_ = 0.19 e Å^−^^3^
0 restraints	Δρ_min_ = −0.27 e Å^−^^3^

### Special details

**Table:**

Geometry. All e.s.d.'s (except the e.s.d. in the dihedral angle between two l.s. planes) are estimated using the full covariance matrix. The cell e.s.d.'s are taken into account individually in the estimation of e.s.d.'s in distances, angles and torsion angles; correlations between e.s.d.'s in cell parameters are only used when they are defined by crystal symmetry. An approximate (isotropic) treatment of cell e.s.d.'s is used for estimating e.s.d.'s involving l.s. planes.
Refinement. Refinement of *F*^2^ against ALL reflections. The weighted *R*-factor *wR* and goodness of fit *S* are based on *F*^2^, conventional *R*-factors *R* are based on *F*, with *F* set to zero for negative *F*^2^. The threshold expression of *F*^2^ > σ(*F*^2^) is used only for calculating *R*-factors(gt) *etc*. and is not relevant to the choice of reflections for refinement. *R*-factors based on *F*^2^ are statistically about twice as large as those based on *F*, and *R*- factors based on ALL data will be even larger.

### Fractional atomic coordinates and isotropic or equivalent isotropic displacement parameters (Å^2^)

**Table:**

	*x*	*y*	*z*	*U*_iso_*/*U*_eq_	
S1	0.04659 (3)	0.66541 (4)	0.36270 (3)	0.06079 (18)	
O1	−0.25953 (6)	1.15940 (11)	0.28999 (6)	0.0511 (3)	
N1	−0.12211 (7)	1.09436 (12)	0.31028 (6)	0.0392 (3)	
N2	−0.03369 (7)	0.95212 (11)	0.40765 (7)	0.0401 (3)	
N3	−0.04059 (7)	0.74873 (11)	0.42680 (7)	0.0410 (3)	
N4	−0.09869 (7)	0.71981 (11)	0.44558 (7)	0.0402 (3)	
N5	−0.06803 (7)	0.58126 (11)	0.39235 (6)	0.0380 (3)	
C1	−0.21966 (9)	1.22602 (15)	0.26341 (8)	0.0414 (4)	
C2	−0.24588 (10)	1.32048 (17)	0.22721 (9)	0.0531 (5)	
H2A	−0.2916	1.3448	0.2233	0.064*	
C3	−0.20535 (12)	1.37922 (18)	0.19688 (10)	0.0612 (5)	
H3A	−0.2239	1.4422	0.1723	0.073*	
C4	−0.13806 (12)	1.34470 (18)	0.20304 (10)	0.0609 (5)	
H4A	−0.1110	1.3826	0.1816	0.073*	
C5	−0.11006 (10)	1.25299 (16)	0.24133 (9)	0.0502 (4)	
H5A	−0.0636	1.2317	0.2460	0.060*	
C6	−0.14894 (9)	1.19165 (14)	0.27302 (8)	0.0392 (4)	
C7	−0.33410 (10)	1.17746 (19)	0.27037 (11)	0.0604 (5)	
H7A	−0.3564	1.1195	0.2879	0.091*	
H7B	−0.3523	1.1760	0.2241	0.091*	
H7C	−0.3439	1.2490	0.2859	0.091*	
C8	−0.05394 (9)	1.05442 (15)	0.30672 (9)	0.0442 (4)	
H8A	−0.0175	1.1089	0.3266	0.053*	
H8B	−0.0560	1.0466	0.2619	0.053*	
C9	−0.03495 (9)	0.94278 (15)	0.34052 (9)	0.0447 (4)	
H9A	−0.0695	0.8868	0.3187	0.054*	
H9B	0.0112	0.9190	0.3388	0.054*	
C10	−0.10279 (9)	0.98856 (14)	0.41065 (8)	0.0407 (4)	
H10A	−0.1018	0.9943	0.4553	0.049*	
H10B	−0.1385	0.9339	0.3894	0.049*	
C11	−0.12140 (9)	1.10112 (15)	0.37793 (8)	0.0420 (4)	
H11A	−0.1677	1.1245	0.3796	0.050*	
H11B	−0.0869	1.1565	0.4006	0.050*	
C12	−0.00463 (9)	0.85666 (15)	0.44685 (9)	0.0449 (4)	
C13	−0.02030 (9)	0.66608 (14)	0.39407 (8)	0.0419 (4)	
C14	−0.11453 (8)	0.61806 (13)	0.42412 (8)	0.0361 (4)	
C15	−0.06398 (9)	0.47423 (15)	0.36034 (9)	0.0460 (4)	
H15A	−0.0532	0.4888	0.3205	0.055*	
H15B	−0.1098	0.4374	0.3494	0.055*	
C16	−0.00816 (11)	0.39690 (17)	0.40219 (12)	0.0619 (5)	
H16A	−0.0079	0.3279	0.3796	0.093*	
H16B	−0.0187	0.3818	0.4416	0.093*	
H16C	0.0375	0.4319	0.4120	0.093*	
C17	−0.17657 (8)	0.55496 (13)	0.43342 (8)	0.0364 (4)	
C18	−0.23590 (9)	0.54186 (17)	0.36848 (9)	0.0499 (5)	
H18A	−0.2510	0.6151	0.3501	0.060*	
H18B	−0.2181	0.5003	0.3384	0.060*	
C19	−0.29894 (10)	0.4796 (2)	0.37923 (10)	0.0603 (5)	
H19A	−0.3360	0.4702	0.3378	0.072*	
C20	−0.27523 (11)	0.36486 (17)	0.40858 (10)	0.0565 (5)	
H20A	−0.3155	0.3246	0.4142	0.068*	
H20B	−0.2563	0.3216	0.3799	0.068*	
C21	−0.21867 (10)	0.37929 (14)	0.47347 (9)	0.0475 (4)	
H21A	−0.2040	0.3053	0.4924	0.057*	
C22	−0.15471 (9)	0.43877 (14)	0.46354 (8)	0.0410 (4)	
H22A	−0.1357	0.3944	0.4354	0.049*	
H22B	−0.1180	0.4469	0.5047	0.049*	
C23	−0.20662 (11)	0.62237 (15)	0.47925 (10)	0.0507 (5)	
H23A	−0.2210	0.6960	0.4612	0.061*	
H23B	−0.1702	0.6319	0.5204	0.061*	
C24	−0.27071 (11)	0.56134 (16)	0.48944 (11)	0.0560 (5)	
H24A	−0.2893	0.6053	0.5186	0.067*	
C25	−0.24790 (12)	0.44675 (16)	0.51877 (10)	0.0559 (5)	
H25A	−0.2118	0.4550	0.5603	0.067*	
H25B	−0.2881	0.4081	0.5254	0.067*	
C26	−0.32823 (11)	0.54824 (19)	0.42461 (13)	0.0687 (6)	
H26A	−0.3431	0.6214	0.4060	0.082*	
H26B	−0.3691	0.5107	0.4307	0.082*	
H12B	0.0444 (11)	0.8413 (14)	0.4491 (9)	0.041 (5)*	
H12A	−0.0064 (10)	0.8708 (16)	0.4920 (10)	0.047 (5)*	

### Atomic displacement parameters (Å^2^)

**Table:**

	*U*^11^	*U*^22^	*U*^33^	*U*^12^	*U*^13^	*U*^23^
S1	0.0474 (3)	0.0637 (3)	0.0818 (4)	−0.0027 (2)	0.0354 (3)	0.0117 (3)
O1	0.0367 (6)	0.0607 (8)	0.0589 (7)	0.0015 (5)	0.0190 (6)	0.0018 (6)
N1	0.0369 (7)	0.0470 (8)	0.0371 (7)	0.0033 (6)	0.0162 (6)	0.0050 (6)
N2	0.0353 (7)	0.0400 (7)	0.0438 (7)	−0.0084 (6)	0.0102 (6)	0.0071 (6)
N3	0.0369 (7)	0.0382 (7)	0.0488 (8)	−0.0090 (6)	0.0146 (6)	0.0065 (6)
N4	0.0393 (7)	0.0368 (7)	0.0465 (8)	−0.0088 (6)	0.0163 (6)	0.0046 (6)
N5	0.0357 (7)	0.0383 (7)	0.0413 (7)	−0.0050 (5)	0.0139 (6)	0.0039 (6)
C1	0.0408 (8)	0.0461 (9)	0.0374 (8)	0.0006 (7)	0.0119 (7)	−0.0064 (7)
C2	0.0489 (10)	0.0551 (11)	0.0510 (10)	0.0109 (8)	0.0091 (8)	−0.0003 (9)
C3	0.0687 (13)	0.0524 (11)	0.0572 (11)	0.0101 (10)	0.0115 (10)	0.0121 (10)
C4	0.0680 (13)	0.0610 (12)	0.0575 (12)	−0.0006 (10)	0.0249 (10)	0.0158 (10)
C5	0.0469 (10)	0.0566 (11)	0.0510 (10)	0.0035 (8)	0.0206 (8)	0.0090 (9)
C6	0.0403 (8)	0.0435 (9)	0.0340 (8)	0.0006 (7)	0.0116 (6)	−0.0011 (7)
C7	0.0373 (10)	0.0740 (13)	0.0698 (13)	−0.0014 (9)	0.0162 (9)	−0.0158 (11)
C8	0.0399 (9)	0.0523 (10)	0.0457 (9)	0.0031 (7)	0.0208 (7)	0.0087 (8)
C9	0.0384 (9)	0.0479 (9)	0.0513 (10)	0.0024 (7)	0.0188 (7)	0.0068 (8)
C10	0.0394 (8)	0.0461 (9)	0.0380 (8)	−0.0102 (7)	0.0140 (7)	0.0000 (7)
C11	0.0431 (9)	0.0474 (9)	0.0376 (8)	−0.0020 (7)	0.0157 (7)	0.0006 (7)
C12	0.0367 (9)	0.0431 (9)	0.0485 (10)	−0.0124 (7)	0.0038 (7)	0.0081 (8)
C13	0.0344 (8)	0.0443 (9)	0.0453 (9)	−0.0033 (7)	0.0101 (7)	0.0122 (8)
C14	0.0375 (8)	0.0347 (8)	0.0366 (8)	−0.0046 (6)	0.0120 (6)	0.0058 (7)
C15	0.0457 (9)	0.0468 (9)	0.0474 (9)	−0.0047 (8)	0.0171 (8)	−0.0041 (8)
C16	0.0531 (11)	0.0473 (10)	0.0829 (15)	0.0033 (8)	0.0174 (10)	−0.0013 (10)
C17	0.0390 (8)	0.0335 (8)	0.0391 (8)	−0.0079 (6)	0.0154 (7)	0.0025 (7)
C18	0.0422 (9)	0.0618 (11)	0.0441 (9)	−0.0080 (8)	0.0109 (7)	0.0140 (9)
C19	0.0403 (10)	0.0835 (14)	0.0527 (11)	−0.0191 (10)	0.0079 (8)	0.0068 (11)
C20	0.0600 (12)	0.0545 (11)	0.0612 (12)	−0.0278 (9)	0.0280 (9)	−0.0116 (10)
C21	0.0615 (11)	0.0347 (8)	0.0530 (10)	−0.0109 (8)	0.0277 (9)	0.0040 (8)
C22	0.0487 (9)	0.0362 (8)	0.0393 (8)	−0.0065 (7)	0.0151 (7)	0.0033 (7)
C23	0.0579 (11)	0.0366 (9)	0.0684 (12)	−0.0104 (8)	0.0351 (9)	−0.0055 (9)
C24	0.0626 (12)	0.0443 (10)	0.0778 (14)	−0.0117 (9)	0.0464 (11)	−0.0081 (9)
C25	0.0698 (12)	0.0532 (11)	0.0568 (11)	−0.0191 (9)	0.0372 (10)	−0.0017 (9)
C26	0.0457 (11)	0.0653 (13)	0.1038 (18)	−0.0044 (9)	0.0356 (11)	0.0186 (13)

### Geometric parameters (Å, º)

**Table:**

S1—C13	1.6674 (18)	C11—H11B	0.9700
O1—C1	1.369 (2)	C12—H12B	0.98 (2)
O1—C7	1.423 (2)	C12—H12A	1.01 (2)
N1—C6	1.423 (2)	C14—C17	1.508 (2)
N1—C8	1.457 (2)	C15—C16	1.512 (3)
N1—C11	1.472 (2)	C15—H15A	0.9700
N2—C12	1.434 (2)	C15—H15B	0.9700
N2—C10	1.457 (2)	C16—H16A	0.9600
N2—C9	1.459 (2)	C16—H16B	0.9600
N3—C13	1.349 (2)	C16—H16C	0.9600
N3—N4	1.3790 (19)	C17—C23	1.538 (2)
N3—C12	1.472 (2)	C17—C22	1.539 (2)
N4—C14	1.305 (2)	C17—C18	1.545 (2)
N5—C13	1.378 (2)	C18—C19	1.532 (2)
N5—C14	1.383 (2)	C18—H18A	0.9700
N5—C15	1.470 (2)	C18—H18B	0.9700
C1—C2	1.383 (3)	C19—C20	1.524 (3)
C1—C6	1.413 (2)	C19—C26	1.529 (3)
C2—C3	1.378 (3)	C19—H19A	0.9800
C2—H2A	0.9300	C20—C21	1.520 (3)
C3—C4	1.362 (3)	C20—H20A	0.9700
C3—H3A	0.9300	C20—H20B	0.9700
C4—C5	1.385 (3)	C21—C25	1.519 (3)
C4—H4A	0.9300	C21—C22	1.525 (2)
C5—C6	1.390 (2)	C21—H21A	0.9800
C5—H5A	0.9300	C22—H22A	0.9700
C7—H7A	0.9600	C22—H22B	0.9700
C7—H7B	0.9600	C23—C24	1.538 (2)
C7—H7C	0.9600	C23—H23A	0.9700
C8—C9	1.513 (2)	C23—H23B	0.9700
C8—H8A	0.9700	C24—C25	1.519 (3)
C8—H8B	0.9700	C24—C26	1.526 (3)
C9—H9A	0.9700	C24—H24A	0.9800
C9—H9B	0.9700	C25—H25A	0.9700
C10—C11	1.513 (2)	C25—H25B	0.9700
C10—H10A	0.9700	C26—H26A	0.9700
C10—H10B	0.9700	C26—H26B	0.9700
C11—H11A	0.9700		
			
C1—O1—C7	117.79 (15)	N5—C14—C17	127.20 (14)
C6—N1—C8	115.28 (13)	N5—C15—C16	112.40 (15)
C6—N1—C11	114.41 (13)	N5—C15—H15A	109.1
C8—N1—C11	110.33 (13)	C16—C15—H15A	109.1
C12—N2—C10	114.93 (14)	N5—C15—H15B	109.1
C12—N2—C9	114.58 (15)	C16—C15—H15B	109.1
C10—N2—C9	109.92 (13)	H15A—C15—H15B	107.9
C13—N3—N4	112.65 (13)	C15—C16—H16A	109.5
C13—N3—C12	126.97 (15)	C15—C16—H16B	109.5
N4—N3—C12	120.21 (15)	H16A—C16—H16B	109.5
C14—N4—N3	104.94 (13)	C15—C16—H16C	109.5
C13—N5—C14	108.06 (14)	H16A—C16—H16C	109.5
C13—N5—C15	120.98 (14)	H16B—C16—H16C	109.5
C14—N5—C15	130.96 (13)	C14—C17—C23	108.67 (13)
O1—C1—C2	123.40 (16)	C14—C17—C22	111.92 (13)
O1—C1—C6	116.34 (15)	C23—C17—C22	108.02 (14)
C2—C1—C6	120.23 (17)	C14—C17—C18	110.51 (13)
C3—C2—C1	121.06 (18)	C23—C17—C18	108.05 (15)
C3—C2—H2A	119.5	C22—C17—C18	109.55 (13)
C1—C2—H2A	119.5	C19—C18—C17	109.64 (14)
C4—C3—C2	119.73 (19)	C19—C18—H18A	109.7
C4—C3—H3A	120.1	C17—C18—H18A	109.7
C2—C3—H3A	120.1	C19—C18—H18B	109.7
C3—C4—C5	119.8 (2)	C17—C18—H18B	109.7
C3—C4—H4A	120.1	H18A—C18—H18B	108.2
C5—C4—H4A	120.1	C20—C19—C26	109.87 (17)
C4—C5—C6	122.32 (18)	C20—C19—C18	109.84 (17)
C4—C5—H5A	118.8	C26—C19—C18	109.18 (18)
C6—C5—H5A	118.8	C20—C19—H19A	109.3
C5—C6—C1	116.69 (16)	C26—C19—H19A	109.3
C5—C6—N1	123.09 (15)	C18—C19—H19A	109.3
C1—C6—N1	120.10 (15)	C21—C20—C19	109.41 (15)
O1—C7—H7A	109.5	C21—C20—H20A	109.8
O1—C7—H7B	109.5	C19—C20—H20A	109.8
H7A—C7—H7B	109.5	C21—C20—H20B	109.8
O1—C7—H7C	109.5	C19—C20—H20B	109.8
H7A—C7—H7C	109.5	H20A—C20—H20B	108.2
H7B—C7—H7C	109.5	C25—C21—C20	110.15 (17)
N1—C8—C9	111.00 (14)	C25—C21—C22	110.15 (15)
N1—C8—H8A	109.4	C20—C21—C22	109.18 (15)
C9—C8—H8A	109.4	C25—C21—H21A	109.1
N1—C8—H8B	109.4	C20—C21—H21A	109.1
C9—C8—H8B	109.4	C22—C21—H21A	109.1
H8A—C8—H8B	108.0	C21—C22—C17	110.05 (14)
N2—C9—C8	110.21 (15)	C21—C22—H22A	109.7
N2—C9—H9A	109.6	C17—C22—H22A	109.7
C8—C9—H9A	109.6	C21—C22—H22B	109.7
N2—C9—H9B	109.6	C17—C22—H22B	109.7
C8—C9—H9B	109.6	H22A—C22—H22B	108.2
H9A—C9—H9B	108.1	C17—C23—C24	110.33 (14)
N2—C10—C11	109.91 (13)	C17—C23—H23A	109.6
N2—C10—H10A	109.7	C24—C23—H23A	109.6
C11—C10—H10A	109.7	C17—C23—H23B	109.6
N2—C10—H10B	109.7	C24—C23—H23B	109.6
C11—C10—H10B	109.7	H23A—C23—H23B	108.1
H10A—C10—H10B	108.2	C25—C24—C26	109.76 (16)
N1—C11—C10	110.43 (14)	C25—C24—C23	109.57 (17)
N1—C11—H11A	109.6	C26—C24—C23	109.29 (17)
C10—C11—H11A	109.6	C25—C24—H24A	109.4
N1—C11—H11B	109.6	C26—C24—H24A	109.4
C10—C11—H11B	109.6	C23—C24—H24A	109.4
H11A—C11—H11B	108.1	C21—C25—C24	109.14 (15)
N2—C12—N3	116.71 (13)	C21—C25—H25A	109.9
N2—C12—H12B	113.0 (11)	C24—C25—H25A	109.9
N3—C12—H12B	103.6 (10)	C21—C25—H25B	109.9
N2—C12—H12A	108.6 (11)	C24—C25—H25B	109.9
N3—C12—H12A	106.1 (11)	H25A—C25—H25B	108.3
H12B—C12—H12A	108.5 (15)	C24—C26—C19	109.21 (16)
N3—C13—N5	103.80 (14)	C24—C26—H26A	109.8
N3—C13—S1	128.57 (13)	C19—C26—H26A	109.8
N5—C13—S1	127.63 (14)	C24—C26—H26B	109.8
N4—C14—N5	110.55 (13)	C19—C26—H26B	109.8
N4—C14—C17	122.24 (15)	H26A—C26—H26B	108.3
			
C13—N3—N4—C14	−0.07 (17)	N3—N4—C14—N5	0.16 (17)
C12—N3—N4—C14	−175.71 (14)	N3—N4—C14—C17	−178.86 (14)
C7—O1—C1—C2	10.9 (2)	C13—N5—C14—N4	−0.20 (18)
C7—O1—C1—C6	−167.17 (15)	C15—N5—C14—N4	179.95 (15)
O1—C1—C2—C3	−174.35 (18)	C13—N5—C14—C17	178.76 (15)
C6—C1—C2—C3	3.7 (3)	C15—N5—C14—C17	−1.1 (3)
C1—C2—C3—C4	−0.7 (3)	C13—N5—C15—C16	80.4 (2)
C2—C3—C4—C5	−2.0 (3)	C14—N5—C15—C16	−99.8 (2)
C3—C4—C5—C6	1.7 (3)	N4—C14—C17—C23	−8.7 (2)
C4—C5—C6—C1	1.2 (3)	N5—C14—C17—C23	172.44 (16)
C4—C5—C6—N1	177.19 (17)	N4—C14—C17—C22	−127.92 (16)
O1—C1—C6—C5	174.34 (15)	N5—C14—C17—C22	53.2 (2)
C2—C1—C6—C5	−3.8 (2)	N4—C14—C17—C18	109.70 (18)
O1—C1—C6—N1	−1.8 (2)	N5—C14—C17—C18	−69.2 (2)
C2—C1—C6—N1	−179.97 (15)	C14—C17—C18—C19	−178.89 (16)
C8—N1—C6—C5	−7.3 (2)	C23—C17—C18—C19	−60.1 (2)
C11—N1—C6—C5	122.18 (18)	C22—C17—C18—C19	57.4 (2)
C8—N1—C6—C1	168.57 (15)	C17—C18—C19—C20	−58.9 (2)
C11—N1—C6—C1	−61.93 (19)	C17—C18—C19—C26	61.6 (2)
C6—N1—C8—C9	−172.41 (14)	C26—C19—C20—C21	−59.0 (2)
C11—N1—C8—C9	56.12 (19)	C18—C19—C20—C21	61.1 (2)
C12—N2—C9—C8	−169.87 (13)	C19—C20—C21—C25	59.6 (2)
C10—N2—C9—C8	58.92 (17)	C19—C20—C21—C22	−61.5 (2)
N1—C8—C9—N2	−57.44 (18)	C25—C21—C22—C17	−60.62 (19)
C12—N2—C10—C11	169.35 (14)	C20—C21—C22—C17	60.46 (19)
C9—N2—C10—C11	−59.63 (17)	C14—C17—C22—C21	178.67 (14)
C6—N1—C11—C10	171.40 (13)	C23—C17—C22—C21	59.08 (18)
C8—N1—C11—C10	−56.68 (17)	C18—C17—C22—C21	−58.40 (18)
N2—C10—C11—N1	58.53 (17)	C14—C17—C23—C24	179.37 (15)
C10—N2—C12—N3	69.0 (2)	C22—C17—C23—C24	−59.0 (2)
C9—N2—C12—N3	−59.8 (2)	C18—C17—C23—C24	59.4 (2)
C13—N3—C12—N2	100.2 (2)	C17—C23—C24—C25	60.2 (2)
N4—N3—C12—N2	−84.8 (2)	C17—C23—C24—C26	−60.1 (2)
N4—N3—C13—N5	−0.05 (17)	C20—C21—C25—C24	−60.16 (19)
C12—N3—C13—N5	175.23 (14)	C22—C21—C25—C24	60.3 (2)
N4—N3—C13—S1	179.88 (12)	C26—C24—C25—C21	60.3 (2)
C12—N3—C13—S1	−4.8 (2)	C23—C24—C25—C21	−59.7 (2)
C14—N5—C13—N3	0.14 (17)	C25—C24—C26—C19	−60.0 (2)
C15—N5—C13—N3	−179.98 (13)	C23—C24—C26—C19	60.2 (2)
C14—N5—C13—S1	−179.79 (12)	C20—C19—C26—C24	59.2 (2)
C15—N5—C13—S1	0.1 (2)	C18—C19—C26—C24	−61.3 (2)

### Hydrogen-bond geometry (Å, º)

Cg is the centroid of the C1–C6 benzene ring.

**Table:**

*D*—H···*A*	*D*—H	H···*A*	*D*···*A*	*D*—H···*A*
C11—H11*A*···O1	0.97	2.26	2.903 (2)	123
C18—H18*A*···*Cg*^i^	0.97	2.81	3.748 (2)	162

Symmetry code: (i) *x*−1, −*y*−1, *z*−1/2.
